# Oral Contraceptive Use, Micronutrient Deficiency, and Obesity among Premenopausal Females in Korea: The Necessity of Dietary Supplements and Food Intake Improvement

**DOI:** 10.1371/journal.pone.0158177

**Published:** 2016-06-27

**Authors:** Boyoung Park, Jeongseon Kim

**Affiliations:** 1 Department of Cancer Control and Policy, Graduate School of Cancer Science and Policy, National Cancer Center, Goyang-si, Korea; 2 Cancer Early Detection Branch, Division of Cancer Management Policy, National Cancer Control Institute, National Cancer Center, Goyang-si, Korea; 3 Molecular Epidemiology Branch, Division of Cancer Epidemiology and Prevention, Research Institute, National Cancer Center, Goyang-si, Korea; Institute for Health & the Environment, UNITED STATES

## Abstract

This study addressed the associations between oral contraceptive (OC) use and obesity as measured by recording the body mass index (BMI) of premenopausal females, and possible interactions with micronutrient intake were considered. A group of 39,189 premenopausal females aged 35–59 were included in the analysis; they were in the Health Examinee cohort. Participant BMIs were calculated from anthropometric measurements, and females with a BMI≥25kg/m^2^ were considered obese. Individual OC use, age at first OC use, duration of OC use, nutrient intake, and other covariates were measured with a structured questionnaire. A multivariate logistic regression with an interaction term was applied to identify the odds ratio (OR) and 95% confidence intervals (CI) between OC use and obesity along with consideration of micronutrient intake interactions. OC use is associated with an increased risk of obesity (OR = 1.12, 95% CI = 1.04–1.20), and females who used OCs for more than 6 months over their lifetimes were more likely to be obese (OR = 1.15, 95% CI = 1.01–1.32) compared with those who used OCs for <6 months. There were interaction effects between phosphorus, potassium, vitamin A, vitamin B_1_, vitamin B_2_, niacin, vitamin C intake and total duration of OC use on being obesity (P-value<0.05). When stratified by micronutrient intake, the associations between total OC use duration and obesity were present only among those with calcium, phosphorus, potassium, vitamin A, B_1_, B_2_, C, niacin, and folate intakes below the recommended levels. Efforts to estimate nutrient intake and prevent micronutrient depletion with supplements or food should be considered by clinicians for females who take OC for a long period.

## Introduction

Contraception use by females in their childbearing years and the choice of having safe, effective, affordable and acceptable contraceptive methods as part of family planning are important dimensions of reproductive health. Oral contraceptives (OCs) are currently the most commonly used contraception method in developed countries. In developing countries, female sterilization and intrauterine devices are the two most commonly used contraceptive methods, accounting for 58% of all contraceptive use [1]. However, in developed countries in Asia such as Japan and the Republic of Korea, the prevalence of OC use is lower than it is in other Western countries [1], suggesting that there are different preferred methods depending on the region.

In Western countries, adherence to OC use is poor, with a 6-month discontinuation rate of more than 50% [2, 3]; side effects are the most common reason for poor compliance [2, 4, 5]. Although previous longitudinal studies and randomized controlled trials have shown that OCs do not have an effect on weight change [6, 7], and a recent review by Cochrane suggested that OC use did not significantly affect weight [8], weight gain is frequently reported as a side effect of OC use [9]. The OC discontinuation rate among the females who gained weight from OCs was 40% higher compared to the females who did not gain weight [2].

Another potential effect of OC use is higher micronutrient requirements. Females who used OCs showed lower blood nutrient levels compared with non-users, suggesting that they may need higher amounts of vitamins and minerals. In addition, an association between micronutrient deficiencies and obesity has been suggested to occur via changes in leptin concentrations or increases in the inflammatory response [10–13]. Therefore, OC use and micronutrient deficiency and micronutrient deficiency and obesity are correlated, and interplay between OC use and micronutrient intake in the effect on obesity seems plausible.

In this study, we investigated possible associations between OC use and obesity as measured by recording the body mass indexes (BMI) of premenopausal females in health examinee-based samples in Korea. In particular, the possible interactions between OC use and micronutrient intake in association with obesity were examined.

## Materials and Methods

### Data source and study population

Data from the Health Examinee (HEXA) cohort was part of the Korea Genome Epidemiology Study (KoGES), which is an ongoing cohort study that was started in 2001 to investigate the effects of various factors and their interactions on the development of chronic diseases in the Korean population, and it was used in this study. Health examinees from health examination centers in 14 urban areas around Korea were recruited from 2004–2013, and the baseline survey and measurement information was analyzed. The interviewers gave information about the HEXA cohort, and informed consents were obtained from health examinees who agreed to participate. All participants completed a questionnaire including their past medical history, sociodemographic and behavioral characteristics, reproductive factors, and a validated food frequency questionnaire, which assessed the intake of 106 food items, was administered by the interviewers. Daily nutrient intake was estimated using the sum of the nutrient intake of each food item [14, 15]. In addition, information from physical examinations including anthropometric measurements and biochemical measurements with a fasting blood sample that was part of a routine process was obtained from all participants. Details of the KoGES and HEXA cohort are described elsewhere [16] or at the Korea National Institute of Health website (http://www.nih.go.kr/NIH/eng/main.jsp).

Among the 111,592 female participants aged 35–73, 62,640 had stopped menstruating for more than 12 months, 2,716 had stopped menstruating for approximately 3 months, 9,440 were premenopausal with an irregular cycle, 30,004 were premenopausal with a regular cycle, and 6,792 had missing information about their menstruation. Among the 39,444 premenopausal females, 39,189 females under 60 were included in the analysis. The Institutional Review Board of the National Cancer Center approved this study protocol, which was in compliance with the Declaration of Helsinki (IRB No: NCC2014-0098).

### Variables

For the obesity index, participant BMIs were calculated as weight (kg)/height^2^ (m^2^) by using anthropometric measurements, and females with a BMI ≥25 kg/m^2^ were considered obese according to the World Health Organization’s revised guidelines for Asians [17, 18]. Individual OC use, age at first OC use, and duration of OC use were assessed by a questionnaire. OC use was categorized as never use and use. The age at first OC use and the total duration of OC use were used to calculate the median (<28 years old and ≥28 years old) or quartile values (<3months, 3–6 months, 6–12 months, and >12 months) in the premenopausal females included in this study. The other covariates included age (in 1 year increments), smoking status (never, ex-smoker, or current smoker), drinking status (never, ex-drinker, or current drinker), regular exercise (none, < 150 minutes/week, or ≥ 150 minutes/week) based on current Europe [19], and World Health Organization physical activity guidelines [20], educational attainment (< high school, ≥ college), monthly household income (<$3000/month, ≥$3000/month), marital status (married, single/divorced/widowed), occupation (unemployed/housewife, white collar job, blue collar job), parity (nulliparous, 1–2, 3 or more), and total energy intake (kcal/day, <1400, 1400–1699, 1700–2049, ≥ 2050 as quartile in the included subjects).

Among the assessed nutrients, we included calcium, phosphorus, potassium, vitamin A, vitamin B_1_, vitamin B_2_, niacin, vitamin C and folate because the recommended daily intake level or adequate intake level of these nutrients have been identified for Koreans.

### Statistical analysis

The sociodemographic and health behavioral characteristics of those who never used OCs and those who did were compared by using a chi-square test or a *t*-test. To identify the association between OC use and obesity, we conducted a multivariate logistic regression analysis to estimate the odds ratios (ORs) and 95% confidence intervals (CI) by OC use, age at first OC use, and duration of OC use, and the values were adjusted for age, smoking status, drinking status, regular exercise, educational attainment, monthly household income, marital status, occupation, parity, and total energy intake.

To identify the interaction effects between age of first OC use, duration of OC use and micronutrient intake in association with obesity, the interactions were assessed by using multivariate logistic regression with interaction terms. In addition, the association between the age at first OC use, the duration of OC use and obesity was stratified by micronutrient intake (less than recommended level, recommended level or more). All statistical analyses were performed with SAS software ver. 9.1 (SAS Institute, Cary, NC, USA).

## Results

Of the 39,189 females, 5,508 (16.1%) had some experience in using OC including 5,291 past users and 217 current users. The sociodemographic and health behavioral characteristics of people who used OCs and those who never did are shown in Table 1. The females who took OCs were older and had lower rates of education beyond high school, increased tobacco or alcohol use and engaged in exercise less regularly. They reported lower household incomes, and being unmarried. They were more likely to be engaged in blue collar work. The proportion of obese females was higher in OC users (21.8% in never-users compared with 25.2% in users, P-value <0.001). There were no significant differences in energy intake distribution between the two groups. Among the OC users, the median age at first OC use was 28 years old and the quartile ranges for the duration of OC use were <3 months, 3–6 months, 6–12 months and >12 months.

**Table 1:** Comparison of basic characteristics between oral contraceptive users and nonusers.

Characteristics	Nonusers (n = 33681)	Oral contraceptive users (n = 5508)	P-value ^a^
	n (%)	n (%)	
Age, mean (95% CI)	45.0(44.9–45.0)	45.4 (45.3–45.5)	<0.001
Smoking status			
Never	32323 (96.0)	5046 (91.6)	<0.001
Ex-smoker	405 (1.2)	156 (2.8)	
Current smoker	799 (2.4)	281 (5.1)	
Missing	154 (0.5)	25 (0.5)	
Drinking status			
Never	19291 (57.3)	2612 (47.4)	<0.001
Ex-drinker	692 (2.1)	161 (2.9)	
Current drinker	13610 (40.4)	2720 (49.4)	
Missing	88 (0.3)	15 (0.3)	
Regular exercise			
No	17610 (52.3)	2844 (51.6)	0.020
< 150 minutes/week	4223 (12.5)	704 (12.8)	
≥ 150 minutes/week	10912 (32.4)	1766 (32.1)	
Missing	936 (2.8)	194 (3.5)	
Educational attainment			
< High school	20532 (61.0)	3983 (72.3)	<0.001
≥ College	12882 (38.2)	1458 (26.5)	
Missing	267 (0.8)	67 (1.2)	
Household income			
< $3000/month	12137 (36.0)	2322 (42.2)	<0.001
≥ $3000/month	18088 (53.7)	2487 (45.1)	
Missing	3456 (10.3)	699 (12.7)	
Marital status			
Single, divorced, widow	3215 (9.5)	684 (12.4)	<0.001
Married	30400 (90.3)	4808 (87.3)	
Missing	66(0.2)	16 (0.3)	
Occupation			
Unemployed, housewife	16383 (48.6)	2677 (48.6)	<0.001
White color job	7437 (22.1)	891 (16.2)	
Blue color job	9385 (27.9)	1861 (33.8)	
Missing	476 (1.4)	79 (1.4)	
Parity			
Nullipara	402 (1.2)	119 (2.2)	<0.001
1–2	26821 (79.5)	4382 (79.6)	
≥ 3	4759 (14.1)	865 (15.7)	
Missing	1699 (5.0)	142 (2.5)	
Energy intake (kcal/day)			
<1400	8440 (25.1)	1460 (26.5)	0.076
1400–1699	8073 (24.0)	1263 (22.9)	
1700–2049	8479 (25.2)	1344 (24.4)	
≥ 2050	8363 (24.8)	1379 (25.0)	
Missing	326 (1.0)	62 (1.1)	
Body mass index (kg/m^2^)			
< 25	26214 (77.8)	4097 (74.4)	<0.001
≥ 25	7338 (21.8)	1390 (25.2)	
Missing	129 (0.4)	21 (0.4)	
Age at first OC use			
<28	-	2607 (47.3)	
≥28	-	2172 (39.4)	
Missing	-	729 (13.3)	
Total duration of OC use (months)			
<3 months	-	1572 (28.5)	
3–6 months	-	1187 (21.6)	
6–12 months	-	987 (17.9)	
>12 months	-	1741 (31.6)	
Missing	-	21 (0.4)	

^a^ Chi-square test or t-test results

CI: confidence intervals; OC: oral contraceptives

OC use in females was associated with obesity (OR = 1.12, 95% CI = 1.04–1.20). In addition, age increment, being engaged in blue collar work, higher parity, and higher energy intake were associated with increased obesity, while regular exercise and higher educational attainment and household income decreased the odds of being obese (Table 2).

**Table 2:** Multivariate logistic regression for the associated factors with obesity (body mass index ≥25kg/m^2^).

	BMI<25kg/m	BMI> = 25kg/m^2^	OR (95% CI)^a^
	n (%)	n (%)	
Age			
1 year increment, mean (SD)	44.9 (4.1)	45.6 (4.2)	1.03 (1.03–1.04)
Smoking status			
Never	28094 (95.8)	8323 (95.8)	1
Ex-smoker	427 (1.4)	130 (1.5)	1.13 (0.92–1.39)
Current smoker	839 (2.8)	238 (2.7)	0.96 (0.82–1.11)
Drinking status			
Never	16913 (56.0)	4901 (56.3)	1
Ex-drinker	641 (2.1)	206 (2.4)	1.13 (0.96–1.33)
Current drinker	12672 (41.9)	3603 (41.4)	1.00 (0.95–1.05)
Regular exercise			
No	15629 (53.1)	4745 (55.9)	1
< 150 minutes/week	3856 (13.1)	1050 (12.4)	0.92 (0.85–0.99)
≥ 150 minutes/week	9949 (33.8)	2690 (31.7)	0.87 (0.83–0.93)
Educational attainment, N (%)			
< High school	18137 (60.3)	6307 (73.1)	1
≥ College	11939 (39.7)	2325 (26.9)	0.67 (0.63–0.71)
Monthly household income, N (%)			
<$3000/month	10689 (39.3)	3740 (48.6)	1
≥$3000/month	16535 (60.7)	3958 (51.4)	0.79 (0.75–0.83)
Marital status			
Married	3007 (9.9)	875 (10.0)	1
Single, divorced, widow	27240 (90.1)	7836 (90.0)	1.01 (0.92–1.11)
Occupation			
Unemployed, housewife	14730 (49.3)	4255 (49.5)	1
White color job	6834 (22.9)	1450 (16.9)	0.95 (0.89–1.02)
Blue color job	8336 (27.9)	2883 (33.6)	1.08 (1.02–1.14)
Parity			
Nullipara	433 (1.5)	88 (1.1)	1
1–2	24358 (84.6)	6727 (80.1)	1.30 (1.03–1.65)
≥ 3	4017 (13.9)	1585 (18.9)	1.70 (1.34–2.17)
Energy intake (kcal/day)			
<1400	7842 (26.1)	2024 (23.5)	1
1400–1699	7257 (24.2)	2045 (23.7)	1.11 (1.03–1.19)
1700–2049	7599 (25.3)	2187 (25.4)	1.16 (1.08–1.24)
≥ 2050	7341 (24.4)	2366 (27.4)	1.33 (1.24–1.42)
Oral contraceptive use			
Never	26214 (86.5)	7338 (84.1)	1
Ever	4097 (13.5)	1390 (15.9)	1.12 (1.04–1.20)

CI: Standard deviation

^a^ Adjusted for age, smoking status, drinking status, regular exercise, educational attainment, monthly household income, marital status, parity, occupation, and energy intake

Among the OC users, age at first OC use was not significantly associated with obesity. However, those who used OCs for longer periods showed an independent increased association with obesity (OR = 1.22, 95%, CI = 1.02–1.46 for those who used OCs for 3–6 months; OR = 1.22, 95%, CI = 1.01–1.48 for those used OCs for 6–12 months, compared with those who used OCs for less than 3 months), although the results for participants who used OCs for more than 12 months were not significant (Table 3). When applied median value, females who used OCs for 6 months or more were more likely to be obese (OR = 1.15, 95%, CI = 1.01–1.32) compared with those who used OC <6 months (data not shown).

**Table 3:** Associations between age at first OC use, duration of oral contraceptives use and obesity (body mass index ≥25kg/m^2^) in ever-oral contraceptive users.

	OR (95% CI) ^a^	P-value
Age at first oral contraceptives use		
<28	1	
≥28	0.99 (0.87–1.14)	0.915
Total duration of oral contraceptives use		
<3 months	1	
3–6 months	1.22 (1.02–1.46)	0.031
6–12 months	1.22 (1.01–1.48)	0.036
>12 months	1.15 (0.96–1.34)	0.133

^a^ Adjusted for age, smoking status, drinking status, regular exercise, educational attainment, monthly household income, marital status, parity, occupation, and energy intake

In addition, there were significant interaction effects between phosphorus, potassium, vitamin A, B_1_, B_2_, C, niacin intake and total duration of OC use (P-interaction = 0.044, 0.001, 0.012, 0.027, 0.034, 0.038, and 0.003, respectively. See Table 4). The analyses that were stratified by micronutrient intake showed that OC intake for 3 months or more increased the odds of being obese among those whose intakes of calcium, phosphorus, potassium, vitamin A, vitamin B_1_, vitamin B_2_, niacin, vitamin C, and folate were lower than the recommended levels, yielding odds ratios ranging from 1.22 to 1.70 (Table 4). For those consuming more than the recommended levels of these nutrients, the duration of OC use was not associated with obesity.

**Table 4:** Associations between age at first oral contraceptives use, duration of oral contraceptives use and obesity (body mass index ≥25kg/m^2^) in ever-oral contraceptive users, stratified by micronutrient intake.

	P-interaction	Lower than required value	Upper than required value
		OR (95% CI)^a^	OR (95% CI) ^a^
Age at first oral contraceptives use			
Calcium intake			
<28	0.475	1	1
≥28		0.99 (0.85–1.14)	1.05 (0.72–1.52)
Phosphorus intake			
<28	0.820	1	1
≥28		1.01 (0.86–1.18)	0.95 (0.73–1.22)
Potassium intake			
<28	0.078	1	1
≥28		0.95 (0.82–1.10)	1.27 (0.87–1.85)
Vitamin A intake			
<28	0.839	1	1
≥28		1.01 (0.86–1.18)	0.95 (0.72–1.26)
Vitamin B_1_ intake			
<28	0.385	1	1
≥28		1.06 (0.89–1.26)	0.91 (0.73–1.12)
Vitamin B_2_ intake			
<28	0.475	1	1
≥28		1.03 (0.88–1.21)	0.90 (0.68–1.18)
Niacin intake			
<28	0.352	1	1
≥28		0.94 (0.77–1.14)	1.04 (0.86–1.26)
Vitamin C intake			
<28	0.219	1	1
≥28		0.93 (0.77–1.14)	1.08 (0.89–1.31)
Folate intake			
<28	0.582	1	1
≥28		0.98 (0.85–1.14)	1.06 (0.67–1.68)
Total duration of oral contraceptives use			
Calcium intake			
<3 months	0.083	1	1
3–6 months		1.23 (1.01–1.49)	1.05 (0.65–1.70)
6–12 months		1.30 (1.06–1.59)	0.79 (0.46–1.36)
>12 months		1.24 (1.04–1.48)	0.79 (0.50–1.24)
Phosphorus intake			
<3 months	0.044	1	1
3–6 months		1.26 (1.01–1.56)	1.12 (0.80–1.57)
6–12 months		1.44 (1.16–1.80)	0.80 (0.55–1.15)
>12 months		1.22 (1.10–1.49)	1.02 (0.75–1.39)
Potassium intake			
<3 months	0.001	1	1
3–6 months		1.29 (1.06–1.57)	0.83 (0.56–1.18)
6–12 months		1.41 (1.15–1.73)	0.42 (0.23–0.74)
>12 months		1.25 (1.04–1.49)	0.75 (0.49–1.17)
Vitamin A intake			
<3 months	0.012	1	1
3–6 months		1.34 (1.09–1.65)	0.95 (0.65–1.37)
6–12 months		1.42 (1.14–1.76)	0.76 (0.51–1.12)
>12 months		1.33 (1.10–1.61)	0.75 (0.54–1.05)
Vitamin B_1_ intake			
<3 months	0.027	1	1
3–6 months		1.49 (1.17–1.88)	0.95 (0.72–1.27)
6–12 months		1.51 (1.18–1.92)	0.91 (0.67–1.23)
>12 months		1.31 (1.06–1.62)	0.99 (0.77–1.29)
Vitamin B_2_ intake			
<3 months	0.034	1	1
3–6 months		1.34 (1.09–1.65)	0.91 (0.63–1.31)
6–12 months		1.39 (1.12–1.73)	0.83 (0.56–1.22)
>12 months		1.30 (1.07–1.56)	0.82 (0.59–1.15)
Niacin intake			
<3 months	0.003	1	1
3–6 months		1.70 (1.31–2.22)	0.94 (0.74–1.21)
6–12 months		1.69 (1.28–2.23)	0.93 (0.72–1.20)
>12 months		1.36 (1.07–1.73)	1.05 (0.84–1.31)
Vitamin C intake			
<3 months	0.038	1	1
3–6 months		1.38 (1.06–1.81)	1.96 (0.85–1.39)
6–12 months		1.64 (1.26–2.15)	0.92 (0.70–1.20)
>12 months		1.33 (1.05–1.69)	1.04 (0.83–1.30)
Folate intake			
<3 months	0.161	1	1
3–6 months		1.27 (1.06–1.54)	0.76 (0.41–1.42)
6–12 months		1.29 (1.06–1.66)	0.68 (0.34–1.35)
>12 months		1.22 (1.03–1.45)	0.69 (0.39–1.20)

^a^ Adjusted for age, smoking status, drinking status, regular exercise, educational attainment, monthly household income, marital status, parity, occupation, and energy intake

## Discussion

This study employed baseline information to show that premenopausal females who used OCs had 12% higher odds of being obese, and among OC users, OC use duration of more than 3 months had a positive association with obesity. However, when stratified by micronutrient intake, OC use of more than 3 months had a positive association with obesity among females whose micronutrient intake was not sufficient. For those who took in more than recommended, the duration of OC use was not associated with being obese. Although this study design did not allow for causal inference, the findings suggested that consuming adequate levels of micronutrients may be necessary for OC users, who may also face higher odds of obesity.

Although a direct comparison with previous clinical trials that showed OC use did not affect weight change [6–8] might be difficult, our results showed that OC users had an increased tendency to be obese after controlling for the effects of other factors. Although the distribution of energy intake was not different, OC users exhibited more unhealthy behaviors and lower socioeconomic characteristics, such as smoking, drinking, completing less education, earning lower incomes, being unmarried and working a blue collar job. Thus, differences in unmeasured covariates and health behaviors between OC users and non-users may increase the odds of obesity in OC users. Otherwise, the effect of OCs may be different according to regions or ethnicities when considering that all previous clinical trials included in the Cochrane review were conducted in Western countries, and there was no Asian study included. To identify the causal association, we suggest that future studies target females living in Asia where OC use is not a common contraceptive method.

Several review studies have shown that blood folate, vitamin B_2_, vitamin B_6_, vitamin B_12_, vitamin C, vitamin E, zinc, selenium, and magnesium was lower in OC users in comparison with non-users [13, 21]. It is well-known that nutrient deficiencies in antioxidants, vitamin A, vitamin B complex, vitamin D, calcium, iron, and zinc have been associated with obesity [10, 22]. These deficiencies included low serum levels or lower intake [10]. The increased intake of calcium prevents fatty acid synthesis and promotes lipolysis and lipid oxidation [10, 23]; phosphorus consumption from food compromises adenosine triphosphate production, which regulates energy metabolism and regulates insulin release [24]; vitamin A inhibits adipogenesis, increases fat cell apoptosis, and regulates leptin [10, 25–27]; and vitamin C, an antioxidant, regulates gene expression involved in adipogenesis and glucocorticoid metabolism [10]. Although a direct mechanism linking potassium, B vitamins and obesity is not evident, lower potassium consumption induces insulin resistance and increases metabolic syndrome [28], and several previous studies have shown lower levels of B vitamins in obese people [10]. Considering that OC use may increase micronutrient intake requirements [13], the relationships among total duration of OC use and micronutrient intake in association with obesity observed in this study may be related to unmet micronutrient needs, an intermediately associated factor in the relationship between longer OC use and obesity. In addition, we did not observe significant associations for longer duration OC use among those who ingested micronutrients above the recommended levels. No significant interplay between age at which OCs were started and micronutrient intake on the associations with obesity was observed, suggesting an interaction between cumulative exposure to OCs and higher micronutrient requirements, which may be associated with obesity. Although we adjusted for possible confounding variables, further prospective studies are necessary to understand whether these interactions are causal.

As the authors acknowledge, this is the first study to investigate possible interactions between OC use and micronutrient intake, and the major limitation of this study should be mentioned. This study employed a baseline survey along with measurements from premenopausal females who visited health examination centers, and it used a cross-sectional approach that has an important limitation in the interpretation of causal relations between exposure and outcome. However, it seems unlikely that obesity could be the determinant of OC use, and obesity was measured at the time of study recruitment; OC use reflected both past and current exposure, by including both current and past users. The number of current OC users was too small (217 of 39,189, 0.55%), and thus we could not observe the current OC use effect on obesity. The study subjects were health examinees who went for health check-ups and agreed to participate in the HEXA cohort study. Although the Korean government offers various health screenings to the whole population for free or at low cost to increase accessibility, the participants might be more concerned about their health status than populations who did not receive health examinations. Thus, the generalization of our results to all premenopausal females who ever used OCs should be treated with caution. The prevalence of current OC use among married women aged 15–44 was 2.3% in Korea [29], which was higher than our results (0.55%), but the marital status and age distribution of these two populations were not different, and therefore, a direct comparison might be not possible. Following the 1990s, most OCs in use have been a combination of estrogens and progestins, and a few might contain estrogen alone or progesterone only, but component information was not available. Information on heights and weights was taken by trained health center workers, but OC use and nutrient intake were obtained with questionnaires. Although there is a possibility of some recall bias, misclassification of OC use may not be systematic because we used the baseline information from the cohort study, which is less affected by recall bias [30], and its influence over the true effect would be minimal. The validated food frequency questionnaire, which covered food items that are generally consumed by Koreans [14], was applied for nutrient intake, and thus a bias caused by the nutrition intake measurement also would not be serious. We could not determine whether the source of the nutrients was from food or dietary supplements because daily nutrient intake was calculated using the sum of the nutrient intake for each food item and was not divided by the intake source. We tried to control for the effects of other covariates through multivariate adjustment, but residual confusion from uncontrolled variables would remain. For example, polycystic ovarian syndrome, which has a prevalence in Korean females of 4–6% [31, 32] could be a confounding factor by possibly increasing obesity and OC intake [33]; however, neither the questionnaire nor medical examination assessed each subject’s history of polycystic ovarian syndrome.

Despite these limitations, the strength of this study is that it includes a large number of subjects with a sufficient number of females who have used OCs. Considering the lower prevalence of OC use in Asian females [1], a large study population is needed to obtain enough OC users. Because all the participants were health examinees, we expect that they would have relatively homogenous characteristics compared with population-based samples, and the comparability of the study population would be increased.

In conclusion, females who have taken OCs had higher odds of obesity than never-users, and those who took OC for 3 months or more over their lifetimes had an increased association with obesity. However, the association between total duration of OC use of 3 months or more and obesity was modified by micronutrient intake, and it was only present among those whose micronutrient intakes were below the recommended levels. Considering that these micronutrients play a role not only in obesity but also in many functions used to regulate our multiple metabolic pathways, efforts to increase micronutrient intake should be considered for females taking OCs. Future clinical trials may confirm the effects of the interaction between cumulative OC use and micronutrient intake on obesity, and given the results of this study, dietary recommendations to ensure sufficient micronutrient intake should be considered by clinicians for those who take OCs for longer periods.

## References

[1] United Nations. World Contraceptive Use 2011; Department of Economic and Social Affairs Population Division United Nations 2011 (Available: http://www.un.org/esa/population/publications/contraceptive2011/contraceptive2011.htm) [cited 2015.09.28].

[2] Westhoff CL, Heartwell S, Edwards S, Zieman M, Stuart G, Cwiak C, et al Oral contraceptive discontinuation: do side effects matter? American Journal of Obstetrics and Gynecology. 2007;196(4):412 e1-e7. 17403440 10.1016/j.ajog.2006.12.015 PMC1903378

[3] Oakley D, Sereika S, Bogue E-L. Oral contraceptive pill use after an initial visit to a family planning clinic. Family Planning Perspectives. 1991:150–4. 1936216

[4] Rosenberg MJ, Waugh MS, Meehan TE. Use and misuse of oral contraceptives: risk indicators for poor pill taking and discontinuation. Contraception. 1995;51(5):283–8. 7628201 10.1016/0010-7824(95)00074-k

[5] Rosenberg MJ, Waugh MS. Oral contraceptive discontinuation: a prospective evaluation of frequency and reasons. American Journal of Obstetrics and Gynecology. 1998;179(3):577–82. 9757954 10.1016/s0002-9378(98)70047-x

[6] Berenson AB, Rahman M. Changes in weight, total fat, percent body fat, and central-to-peripheral fat ratio associated with injectable and oral contraceptive use. American Journal of Obstetrics and Gynecology. 2009;200(3):329 e1-e8. 10.1016/j.ajog.2008.12.052 19254592 PMC2853746

[7] De Melo N, Aldrighi J, Faggion D, Reyes V, Souza J, Fernandes C, et al A prospective open-label study to evaluate the effects of the oral contraceptive Harmonet®(gestodene75/EE20) on body fat. Contraception. 2004;70(1):65–71. 15208055 10.1016/j.contraception.2003.10.016

[8] Gallo M, Lopez L, Grimes D, Schulz K, Helmerhorst F. Combination contraceptives: effects on weight (Review). Cochrane Database of Systematic Reviews. 2014;(1):78. 10.1002/14651858.CD003987.pub5 PMC10640873 24477630

[9] Picardo CM, Nichols M, Edelman A, Jensen JT. Women's knowledge and sources of information on the risks and benefits of oral contraception. Journal of the American Medical Women's Association (1972). 2002;58(2):112–6. 12744425

[10] García OP, Long KZ, Rosado JL. Impact of micronutrient deficiencies on obesity. Nutrition Reviews. 2009;67(10):559–72. 10.1111/j.1753-4887.2009.00228.x 19785688

[11] Aasheim ET, Hofsø D, Hjelmesæth J, Birkeland KI, Bøhmer T. Vitamin status in morbidly obese patients: a cross-sectional study. The American Journal of Clinical Nutrition. 2008;87(2):362–9. 18258626 10.1093/ajcn/87.2.362

[12] Kimmons JE, Blanck HM, Tohill BC, Zhang J, Khan LK. Associations between body mass index and the prevalence of low micronutrient levels among US adults. Medscape General Medicine. 2006;8(4):59 17415336 PMC1868363

[13] Palmery M, Saraceno A, Vaiarelli A, Carlomagno G. Oral contraceptives and changes in nutritional requirements. European Review for Medical and Pharmacological Sciences. 2013;17(13):1804–13. 23852908

[14] Ahn Y, Kwon E, Shim J, Park M, Joo Y, Kimm K, et al Validation and reproducibility of food frequency questionnaire for Korean genome epidemiologic study. European Journal of Clinical Nutrition. 2007;61(12):1435–41. 17299477 10.1038/sj.ejcn.1602657

[15] The Korean Nutrition Society (Ed) (2000). Food Composition Table. In: Recommended dietary allowances for Koreans, 7th edition, The Korean Nutrition Society, Seoul.

[16] Health Examinees Study Group. The Health Examinees (HEXA) Study: Rationale, study design and baseline characteristics. Asian Pacific Journal of Cancer Prevention. 2014;16(4):1591–7. 10.7314/apjcp.2015.16.4.1591 25743837

[17] World Health Organization. The Asia-Pacific perspective: redefining obesity and its treatment; 2000. (Available: http://www.wpro.who.int/nutrition/documents/Redefining_obesity/en/) [cited 2016.06.07]

[18] Anuurad E, Shiwaku K, Nogi A, Kitajima K, Enkhmaa B, Shimono K, et al The new BMI criteria for Asians by the regional office for the western pacific region of WHO are suitable for screening of overweight to prevent metabolic syndrome in elder Japanese workers. Journal of Occupational Health. 2003;45(6):335–43. 14676412 10.1539/joh.45.335

[19] Oja P, Bull FC, Fogelholm M, Martin BW. Physical activity recommendations for health: what should Europe do? BMC Public Health. 2010;10(1):1. 20064237 10.1186/1471-2458-10-10 PMC3091541

[20] World Health Organization. Global Recommendations on Physical Activity for Health; 2010 Geneva, Switzerland: World Health Organization. 26180873

[21] Mc Wilson S, Bivins BN, Russell KA, Bailey LB. Oral contraceptive use: impact on folate, vitamin B6, and vitamin B12 status. Nutrition Reviews. 2011;69(10):572–83. 10.1111/j.1753-4887.2011.00419.x 21967158

[22] Via M. The malnutrition of obesity: micronutrient deficiencies that promote diabetes. ISRN Endocrinology. 2012;2012:103472 10.5402/2012/103472 22462011 PMC3313629

[23] Zemel MB. Role of calcium and dairy products in energy partitioning and weight management. The American Journal of Clinical Nutrition. 2004;79(5):907S–12S. 15113738 10.1093/ajcn/79.5.907S

[24] Obeid O. Low phosphorus status might contribute to the onset of obesity. Obesity Reviews. 2013;14(8):659–64. 10.1111/obr.12039 23679666

[25] Xue J-C, Schwarz EJ, Chawla A, Lazar MA. Distinct stages in adipogenesis revealed by retinoid inhibition of differentiation after induction of PPARgamma. Molecular and Cellular Biology. 1996;16(4):1567–75. 8657131 10.1128/mcb.16.4.1567 PMC231142

[26] Kim H, Hausman D, Compton M, Dean R, Martin R, Hausman G, et al Induction of Apoptosis by All-trans-Retinoic Acid and C2-Ceramide Treatment in Rat Stromal–Vascular Cultures. Biochemical and Biophysical Research Communications. 2000;270(1):76–80. 10733907 10.1006/bbrc.2000.2373

[27] Menendez C, Lage M, Peino R, Baldelli R, Concheiro P, Dieguez C, et al Retinoic acid and vitamin D (3) powerfully inhibit in vitro leptin secretion by human adipose tissue. Journal of Endocrinology. 2001;170(2):425–31. 11479138 10.1677/joe.0.1700425

[28] Lee H, Lee J, Hwang S, Kim S, Chin HJ, Han JS, et al Potassium intake and the prevalence of metabolic syndrome: the Korean National Health and Nutrition Examination Survey 2008–2010. PloS One. 2013;8(1):e55106 10.1371/journal.pone.0055106 23372822 PMC3555937

[29] Korea Institute for Health and Social Welfare. The 2012 National Survey on Fertility, Family Health & Welfare in Korea (Korean) [Avaliable: https://www.google.co.kr/url?url=https://www.kihasa.re.kr/html/jsp/share/download_publication.jsp%3Fbid%3D12%26ano%3D1465%26seq%3D1&rct=j&frm=1&q=&esrc=s&sa=U&ved=0CBgQFjAAahUKEwiBpcf0x4XIAhUJjJQKHeMqC3o&usg=AFQjCNF7ceIN2XrGXwbsAJkdx-863ngC2A]. Seoul: Korea Institute for Health and Social Welfare; 2012.

[30] Gordis L. Epidemiology: Elsevier Saunders; 2013.

[31] Byun EK, Kim H-J, Oh J-Y, Hong YS, Sung Y-A. The prevalence of polycystic ovary syndrome in college students from Seoul. Journal of Korean Society of Endocrinology. 2005;20(2):120–6.

[32] Sung Y-A, Kim D-S, Yoo S-J, Baik S-H, Oh J-Y, Lee H-J. The prevalence and phenotypes of polycystic ovary syndrome in Korean women. Endocrine Abstracts. 2010;(22): P486 (Abstract. Available: http://www.endocrine-abstracts.org/ea/0022/eap486.htm) [cited 2016.06.07].

[33] Ehrmann DA. Polycystic ovary syndrome. New England Journal of Medicine. 2005;352(12):1223–36. 15788499 10.1056/NEJMra041536

